# Radiomics Signature to Predict Prognosis in Early-Stage Lung Adenocarcinoma (≤3 cm) Patients with No Lymph Node Metastasis

**DOI:** 10.3390/diagnostics12081907

**Published:** 2022-08-06

**Authors:** Li Zhang, Lv Lv, Lin Li, Yan-Mei Wang, Shuang Zhao, Lei Miao, Yan-Ning Gao, Meng Li, Ning Wu

**Affiliations:** 1 Department of Diagnostic Radiology, National Cancer Center/National Clinical Research Center for Cancer/Cancer Hospital, Chinese Academy of Medical Sciences and Peking Union Medical College, No. 17 Panjiayuan Nanli, Chaoyang District, Beijing 100021, China; 2 Department of Nuclear Medicine, National Cancer Center/National Clinical Research Center for Cancer/Cancer Hospital, Chinese Academy of Medical Sciences and Peking Union Medical College, No. 17 Panjiayuan Nanli, Chaoyang District, Beijing 100021, China; 3 Department of Pathology, National Cancer Center/National Clinical Research Center for Cancer/Cancer Hospital, Chinese Academy of Medical Sciences and Peking Union Medical College, No. 17 Panjiayuan Nanli, Chaoyang District, Beijing 100021, China; 4 GE Healthcare China, Pudong New Town, Shanghai 201200, China; 5 Pediatric Translational Medicine Institute, Shanghai Children’s Medical Center, Shanghai Jiao Tong University School of Medicine, Shanghai 200127, China; 6 State Key Laboratory of Molecular Oncology, Department of Etiology and Carcinogenesis, National Cancer Center/National Clinical Research Center for Cancer/Cancer Hospital, Chinese Academy of Medical Sciences and Peking Union Medical College, No. 17 Panjiayuan Nanli, Chaoyang District, Beijing 100021, China; 7 Department of Diagnostic Radiology, National Cancer Center/National Clinical Research Center for Cancer/Hebei Cancer Hospital, Chinese Academy of Medical Sciences, Langfang 065001, China

**Keywords:** lung adenocarcinoma, computed tomography, radiomics, prognosis

## Abstract

Objectives: To investigate the predictive ability of radiomics signature to predict the prognosis of early-stage primary lung adenocarcinoma (≤3 cm) with no lymph node metastasis (pathological stage I). Materials and Methods: This study included consecutive patients with lung adenocarcinoma (≤3 cm) with no lymph node metastasis (pathological stage I) and divided them into two groups: good prognosis group and poor prognosis group. The association between the radiomics signature and prognosis was explored. An integrative radiomics model was constructed to demonstrate the value of the radiomics signature for individualized prognostic prediction. Results: Six radiomics features were significantly different between the two prognosis groups and were used to construct a radiomics model. On the training and test sets, the area under the receiver operating characteristic curve value of the radiomics model in discriminating between the two groups were 0.946 and 0.888, respectively, and those of the pathological model were 0.761 and 0.798, respectively. A radiomics nomogram combining sex, tumor size and rad-score was built. Conclusion: The radiomics signature has potential utility in estimating the prognosis of patients with pathological stage I lung adenocarcinoma (≤3 cm), potentially enabling a step forward in precision medicine.

## 1. Introduction

Lung cancer ranks first as a cause of cancer-related death worldwide, and adenocarcinoma is the most common histological subtype of lung cancer [1,2]. Low-dose CT (LDCT) screening programs can detect early lung cancer and reduce the mortality of lung cancer [3,4,5,6]. With the advent of the LDCT screening era, peripheral lung nodules, which are defined as rounded opacities measuring up to 3 cm (≤3 cm), are increasingly being detected [7]. The most common type of malignant nodule among them is small lung adenocarcinoma (≤3 cm) with no detectable lymph node metastasis after surgery [3,6,8]. These patients are staged as pathological stage I according to the TNM staging system, most of whom are staged as pathological stage IA (pT_1_N_0_M_0_), and some are staged as pathological stage IB (pT_2_N_0_M_0_) if the visceral pleura is invaded [9]. TNM stage is the single most relevant prognostic factor for recurrence and death after surgery. However, even after complete resection of early lung cancer at the same stage (stage I), patients’ survival time still differs significantly, indicating the urgent need for personalized medicine [10]. For pathological stage I lung adenocarcinoma, the prognosis also varies. Surgery is the standard curative method for stage I lung cancer; however, approximately 27% of patients with stage I lung cancer experience recurrence [11]. Therefore, improved prediction of the prognosis of pathological stage I lung adenocarcinoma in addition to TNM stage may facilitate better decisions on the use of adjuvant chemotherapy or targeted therapy after surgery.

As an emerging field in recent years, radiomics has addressed many medical problems and attracted increased attention. Radiomics is the process of the conversion of medical radiographic images into high-dimensional, quantitative, and mineable data via the high-throughput extraction of large amounts of image-based features, followed by subsequent data analysis for decision support [12,13,14,15]. With the development of machine learning algorithms and the growth of dataset sizes, quantitative radiomics features could potentially serve as a noninvasive biomarker, improve predictive accuracy in oncology, and facilitate individualized treatment [16,17,18,19,20]. Furthermore, some studies have reported associations between radiomic features and underlying gene expression patterns [13,17,21]. These prior studies have suggested that radiomics is a novel and meaningful tool with which could realize precision oncology.

For lung cancer, radiomics has the potential to offer personalized medicine applications, such as differential diagnosis or malignancy prediction [22], histological subtyping [23,24], genetic expression prediction [25,26], tumor stage and distant metastasis prediction [27] and posttreatment prognosis prediction [28,29], using a cost-effective and noninvasive method [30]. Here, we hypothesize that radiomics may be able to detect the heterogeneous internal features of early-stage lung adenocarcinoma and predict prognosis. CT is the most common technique for preoperative evaluation of lung cancer. Therefore, the purpose of the study was to identify the ability of CT-based radiomics to predict the prognosis of pathological stage I lung adenocarcinoma (≤3 cm) in terms of disease-free survival (DFS) outcomes.

## 2. Materials and Methods

### 2.1. Patient Enrollment and Follow-Up

Ethical approval was obtained from our institution for this retrospective analysis, and the requirement to obtain informed consent was waived. This study conducted an evaluation of the institutional database for medical records from 2007 to 2012 to identify patients with histologically confirmed early-stage adenocarcinoma (≤3 cm) with no lymph node metastasis (pathological stage I) who underwent surgical resection with curative intent. The inclusion criteria were as follows: (1) patients with histologically confirmed early-stage adenocarcinoma (≤3 cm) with no lymph node metastasis (pathological stage I) who underwent curative surgical pulmonary resection with negative resection margins, and systematic mediastinal lymph node dissection; (2) patients with enhanced thin-slice CT (1.25 mm) images prior to surgery that could be used in the picture achieving and communication system (PACS); (3) patients with no previous history of other malignant tumors; and (4) patients who were followed up with prognostic information available. The exclusion criteria were as follows: (1) the quality of CT images was too low for observation and analysis owing to breathing artifacts or incorrect scan parameters; and (2) the patient was lost to follow-up. In total, 97 patients were identified.

The patients were classified into two groups (good prognosis group and poor prognosis group) according to their prognosis over follow-up. Within five years, if the patients experienced relapse, defined as tumor recurrence within or immediately adjacent to the treated field, mediastinal relapse, or distant relapse, these patients were classified into the poor prognosis group. If the patients did not experience relapse within 5 years, they were classified into the good prognosis group. DFS was defined as the time from the date of surgery to the date of cancer relapse.

### 2.2. CT Image Acquisition

All 97 of the enrolled patients underwent contrast-enhanced chest CT examination. The spiral CT equipment and scan parameters were as follows: GE Lightspeed Ultra 8-MDCT (120 kV, 230 mAs, reconstruction thickness 1.25 mm, reconstruction spacing 0.8 mm); GE Lightspeed 64-VCT (120 kV, 230 mAs, reconstruction thickness 1.25 mm, reconstruction spacing 0.8 mm); Toshiba Aquilion 64-MDCT (120 kV, 220 mAs, reconstruction thickness 1 mm, spacing 0.8 mm). The enhanced CT scan commenced at 35 s delay after intravenous injection of 85~100 mL of contrast medium (300 mg/mL) using a power injector at a rate of 2.5 mL/s.

### 2.3. Radiomics Segmentation and Feature Extraction

CT images in digital imaging and communications in medicine (DICOM) format were exported and preprocessed by resampling before region of interest (ROI) delineation to eliminate the difference between images with different slice thicknesses. Lesion segmentation was completed by two experienced radiologists majoring in thoracic tumor diagnosis using ITK-SNAP software. The ROI was manually or semiautomatically delineated layer by layer on the lung window image until all the entire tumor was included. The delineated areas of different target lesions were highlighted in the figure with specific colors. Radiomics features were extracted by AK software (Artificial Intelligent Kit, GE Healthcare, Chicago, IL, USA), which conformed with the image biomarker standardization initiative (https://ibsi.readthedocs.io/en/latest/, accessed on 29 May 2022).

### 2.4. Radiomics Analysis and Model Establishment

The dataset was randomly divided into training cohort and test cohort in a 7:3 ratio. All cases in the training cohort were used to train the predictive models, while cases in the test cohort were used to evaluate the performance of the models. The radiomics signatures were standardized in advance and were screened and selected using two methods: maximum relevance minimum redundancy (mRMR) and least absolute shrinkage and selection operator (LASSO). The mRMR method was used to select highly predictive but unrelated features based on the ranking of the correlation redundancy index and retainment of specific features [31]. Then, LASSO regression was used to select the optimal feature subset, evaluate the corresponding coefficients and build a radiomics model [32]. The final prediction model and radiomics score (rad-score) were obtained by logistic linear regression on the selected features using 10-fold cross-validation, i.e., the linear combination was weighted by its respective coefficients and repeated 10 times. The rad-score derived from the significant radiomics features was calculated. The pathological model and radiomics model were constructed. A nomogram was drawn for prognostic effect prediction. The independent predictive risk factors were applied to construct the nomogram.

### 2.5. Statistical Analysis

For clinical and pathological features, continuous data conforming to the normal distribution are expressed as the mean ± standard deviation, otherwise, the median and quartiles are presented. If the quantitative data followed the normal distribution, the *t*-test was used to test the differences between the two groups (the good prognosis group and poor prognosis group); otherwise, the Mann–Whitney U test was used. The chi-square test was used to compare count data, and Fisher’s exact test was used if the assumptions for the chi-square test were not satisfied. All clinical and pathological characteristics of the two groups were compared using the statistical software SPSS version 20.0.

All radiomics statistical analyses in the present study were performed with R 3.5.1 and Python 3.5.6. The multivariate logistic regression method was used to establish the pathological model and the radiomics model. The receiver operating characteristic (ROC) curve and the area under the curve (AUC) of the two datasets were used to determine the discrimination performance of each model. The optimal diagnostic threshold was automatically determined as the point on the ROC curve that maximized the sum of sensitivity and specificity (the highest Youden index). The DeLong test was used to test for significant differences between the ROC curves of the models. Net reclassification index (NRI) analysis was performed to compare the predictions of the radiomics model and the pathological model. In the construction of the individualized nomogram prediction model, clinical variables contributing significantly to prognosis selected using logistic multivariate analysis were also incorporated in addition to the rad-score into a multivariate logistic regression model to establish the nomogram.

In all statistical analyses, *p* values less than 0.05 were considered statistically significant.

## 3. Results

### 3.1. Clinical and Pathological Characteristics

The clinical and pathological characteristics of all enrolled patients and different groups were summarized in Table 1 and Table 2. Sex, smoking, pathological subtype, stage, and tumor size were all significantly different between the two groups.

### 3.2. Radiomics Signature Analysis

**Figure 1:**
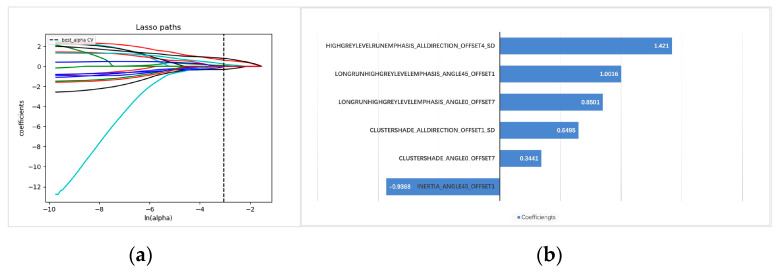
Radiomics signature feature selection and analysis. (**a**) The selection of the tuning parameter in the LASSO model via 10-fold cross-validation based on minimum criteria. (**b**) The radiomics signature contribution bar graph, showing the six ultimately retained radiomics features selected by LASSO and mRMR along with their contributions. The *y*-axis shows the 6 retained radiomics features, and the *x*-axis shows the corresponding LASSO regression coefficients.

For each ROI, 216 radiomics features were extracted, and 17 were selected after the process of dimension reduction (Figure 1). Six statistically significant radiomics features (ClusterShade_AllDirection_offset1_SD, ClusterShade_angle0_offset7, Inertia_angle45_offset1, HighGreyLevelRunEmphasis_AllDirection_offset4_SD, LongRunHighGreyLevelEmphasis_angle0_offset7, and LongRunHighGreyLevelEmphasis_angle45_offset1) remained after multivariate logistic regression analysis (Figure 1). Then, the rad-score was calculated using these six radiomics features as follows: rad-score = 0.6495 × ClusterShade_AllDirection_offset1_SD + −0.3441 × ClusterShade_angle0_offset7 + −0.9368 × Inertia_angle45_offset1 + 1.4210 × HighGreyLevelRunEmphasis_AllDirection_offset4_SD + 0.8501 × LongRunHighGreyLevelEmphasis_angle0_offset7 + 1.0016 × LongRunHighGreyLevelEmphasis_angle45_offset1.

### 3.3. Assessment of the Incremental Value of the Radiomics Model in Predicting Prognosis

**Figure 2:**
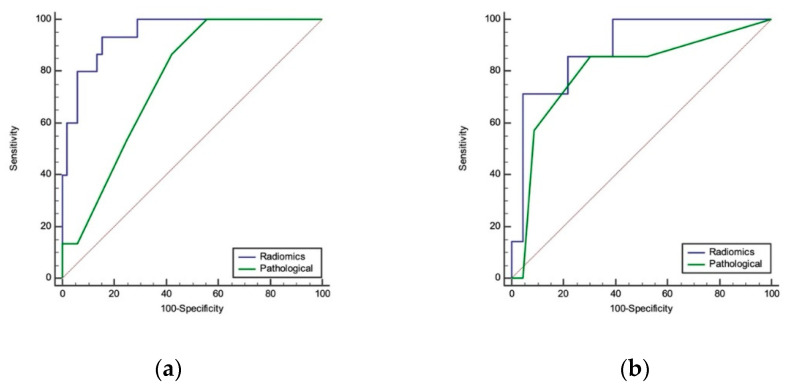
Radiomics and pathological model in the training and test groups. (**a**) The AUC of the radiomics signature model and pathological model in the training group was 0.946 and 0.761, respectively. (**b**) The AUC of the radiomics signature model and pathological model in the test group was 0.888 and 0.798, respectively.

**Figure 3:**
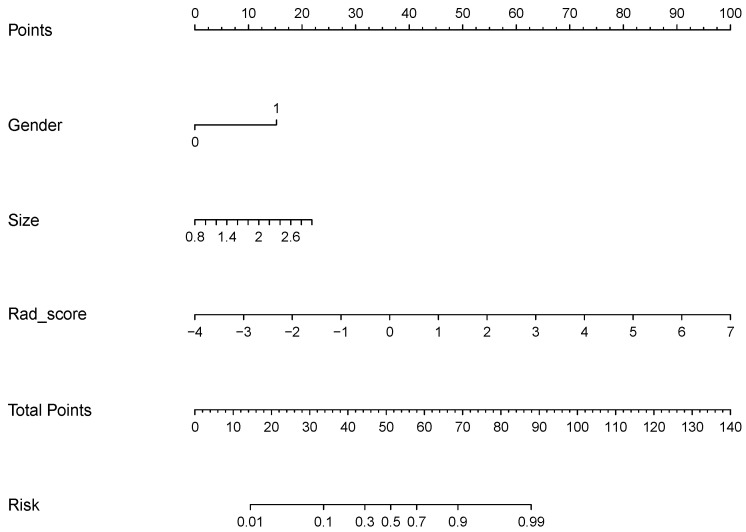
Nomogram for prognosis prediction. The nomogram was developed by integrating the rad-score with sex and tumor size. The probability of rad-score, sex and tumor size can be converted into the points according to the scale at the top of the nomogram by drawing a line straight upward to the “Points” axis. By summing the points for all predictors and locating the final sum on the “Total points” scale, we can predict the probability of recurrence or metastasis on the “Risk” scale.

In the training cohort, the AUCs of the radiomics signature model and pathological (including pathological stage and subtype) model were 0.946 and 0.761 in the training cohort (Figure 2a) and 0.888 and 0.798 in the testing cohort, respectively (Table 3) (Figure 2b). The NRI test showed that the radiomics model was better than the pathological model in both the training and testing cohorts (Table 4). Furthermore, we built a nomogram to predict the prognosis. Among the clinical variables, sex and tumor size were selected using logistic multivariate analysis to establish the nomogram, and the radiomics nomogram combining sex, tumor size and rad-score was presented in Figure 3.

## 4. Discussion

Although the body of radiomics literature in this field has flourished in recent years, to our knowledge, this is the first study about the prognosis prediction of patients with early stage I lung adenocarcinoma (≤3 cm). On CT images, these tumors are described as lung nodules and have attracted increasing attention. A previous study showed that their established radiomics signature was an independent biomarker for the estimation of DFS in patients with early-stage (I or II) non-small-cell lung cancer (NSCLC) [33]. Unlike that study, our study focused specifically on a subset of stage I patients with small lung adenocarcinoma (≤3 cm) and no lymph node metastasis. In this study, we found that the radiomics signature can also predict prognosis in stage I lung adenocarcinoma (≤3 cm) patients.

A total of six radiomics features were identified as significant prognostic imaging biomarkers in our study. The identified signature consisted of the following features: Cluster Shade_All Direction, Cluster Shade_angle 0, Inertia_angle 45, High Grey Level Run Emphasis_All Direction, Long Run High Grey Level Emphasis_angle 0, and Long Run High Grey Level Emphasis_angle 45. These radiomics features mainly involve imaging texture information instead of shape information. Furthermore, this study aimed to establish a radiomics model to predict the prognosis of stage IA lung carcinoma. A total of 6 kinds of radiomics features were selected using a logistic process. The AUCs of the radiomics model were all greater than those of the pathological model (including pathological stage and subtype) in both the training group (0.946 vs. 0.761) and the testing group (0.888 vs. 0.798). The NRI test also showed that the radiomics model was better than the pathological model in both the training cohort and the test cohort. Furthermore, we built an individual nomogram to predict prognosis. The nomogram was developed by integrating the rad-score with two significant clinical features (sex and tumor size). The probability of each predictor can be converted into the points according to the scale at the top of the nomogram by drawing a line straight upward to the “Points” axis. By summing the points for all predictors and locating the final sum on the “Total points” scale, we can predict the probability of recurrence or metastasis on the “Risk” scale at the bottom of the nomogram for individual patients with stage I lung adenocarcinoma (≤3 cm).

Tumor heterogeneity is recognized as an important feature of cancer that may be associated with adverse biological behavior in tumors, and poor prognosis for patients, as cancers with more genomic heterogeneity are more likely to develop resistance to treatment and to metastasize [12,34,35]. Radiomics, which involves the extraction of numerous deep imaging features coupled with appropriate statistical analysis, may reflect the expression of genomic and proteogenomic heterogeneity according to the findings of previous studies [36,37,38]. However, interpreting the complex associations between radiomics signatures and biological processes remains difficult, and further radio-genomics work is needed to establish the biological underpinnings of tumor heterogeneity and identify potential radiomics-biological correlations [39]. Therefore, the results of the current study may suggest that radiomics demonstrated intratumor heterogeneity within early stage I lung adenocarcinoma (≤3 cm), but the relevance between this macroscopic scale and underlying biological scales, such as at the molecular, genetic or cellular levels of lung carcinoma, needs to be investigated in future studies.

TNM stage is the main clinical evidence used to predict the prognosis of cancer. For lung carcinoma, the pathological subtype is an important index related to prognosis [40,41,42,43]. Our study indicated that radiomics signature was also a predictive factor in patients with pathological stage I lung carcinoma (≤3 cm), adding value to the traditional staging system and pathological subtypes for individualized DFS estimation. The radiomics signature can successfully stratify these patients into good prognosis and poor prognosis groups. For patents with pathological stage I lung cancer, assisted chemotherapy or targeted therapy is only recommended for high-risk patients with stage IB. Radiomics may become a screening tool to select high-risk patients with pathological stage I lung cancer, which might enable a step forward in precision medicine.

Our study has several limitations. First, this study is from a single center, and the sample size is relatively small and lacks external validation. Second, the retrospective nature of data collection will cause bias. Multicenter, prospective and large sample size studies are needed to verify the current findings.

In conclusion, the radiomics signature has potential capacity for the prognostic estimation of DFS in patients with early pathological stage I lung adenocarcinoma (≤3 cm), which might enable a step forward in precision medicine.

## Figures and Tables

**Table 1:** Clinical and pathological characteristics of the two groups.

	Total (*n* = 97)	Good Prognosis Group (*n* = 74)	Poor Prognosis Group (*n* = 23)	*p* Value
Age	58.39 ± 10.19	58.08 ± 10.10	59.39 ± 10.63	0.593
Sex				0.002
Female	33 (34.0)	19 (25.7)	14 (60.9)	
Male	64 (66.0)	55 (74.3)	9 (39.1)	
Smoking *				0.006
Yes	21 (22.6)	11 (15.7)	10 (43.5)	
No	72 (77.4)	59 (84.3)	13 (56.5)	
Pathological subtype #				0.002
1	33 (34.0)	31 (41.9)	2 (8.7)	
2	16 (16.5)	14 (18.9)	2 (8.7)	
3	42 (43.3)	25 (33.8)	17 (73.9)	
4	6 (6.2)	4 (5.4)	2 (8.7)	
T stage				<0.001
1a	43 (44.3)	41 (55.4)	2 (8.7)	
1b	11 (11.3)	5 (6.8)	6 (26.1)	
1c	3 (3.1)	2 (2.7)	1 (4.3)	
2a	40 (41.3)	26 (35.1)	14 (60.9)	
Stage				0.029
IA	57 (58.8)	48 (64.9)	9 (39.1)	
IB	40 (41.2)	26 (35.1)	14 (60.9)	
Tumor size (cm)	1.80 ± 0.53	1.72 ± 0.52	2.07 ± 0.49	0.006
DFS (days)	3363.08 ± 106.99	N/A	986.83 ± 165.91	N/A

Note—Data in the table consist of the number of patients first, then the percentage in parentheses. Continuous data conforming to the normal distribution are expressed as the mean ± standard deviation; otherwise, they are expressed as the median and quartiles. * Four patients had unknown smoking status. # 1. AIS or MIA; 2. mural type; 3. acinar or papillary type; 4. micropapillary, solid or variant type.

**Table 2:** Clinical and pathological characteristics of the training and testing cohorts.

Model	Training Cohort (*n* = 67)	Testing Cohort (*n* = 30)	*p* Value
Prognosis			0.953
Good	51 (76.1)	23 (76.7)	
Poor	16 (23.9)	7 (23.3)	
Age	58.96 ± 9.24	57.13 ± 12.13	0.418
Sex			0.713
Female	22 (32.8)	11 (36.7)	
Male	45 (67.2)	19 (63.3)	
Smoking *			0.714
Yes	14 (21.5)	7 (25.0)	
No	51 (78.5)	21 (75.0)	
Pathological subtype #			0.334
1	19 (28.4)	14 (46.7)	
2	13 (19.4)	3 (10)	
3	30 (44.8)	12 (40)	
4	5 (7.4)	1 (3.3)	
T stage			0.054
1a	24 (35.8)	19 (63.3)	
1b	8 (11.9)	3 (10.0)	
1c	2 (3.0)	1 (3.3)	
2a	33 (49.3)	7 (23.4)	
Stage			0.017
IA	34 (50.7)	23 (76.7)	
IB	33 (49.3)	7 (23.3)	
Tumor size (cm)	1.80 ± 0.54	1.80 ± 0.52	0.970
DFS (days)	3154.28 ± 183.86	3469.20 ± 255.61	0.879

Note—Data in the table consist of the number of patients first, then the percentage in parentheses. Continuous data conforming to the normal distribution are expressed as the mean ± standard deviation; otherwise, they are expressed as the median and quartiles. * Four patients had unknown smoking status. # 1. AIS or MIA; 2. mural type; 3. acinar or papillary type; 4. micropapillary, solid or variant type.

**Table 3:** Value of the radiomics signature and pathological models.

Model Type	Training Cohort			Testing Cohort		
	AUC	SE	95% CI	AUC	SE	95% CI
Pathological	0.761	0.0575	0.648 to 0.874	0.798	0.107	0.588 to 1.000
Radiomics	0.946	0.0268	0.894 to 0.999	0.888	0.0675	0.756 to 1.000

**Table 4:** Net reclassification index (NRI) test outcome.

NRI	Training Cohort			Testing Cohort		
	Estimate	Std. Error	Lower to Upper	Estimate	Std. Error	Lower to Upper
Radiomics vs. Pathological model	0.3808	0.1357	0.1116 to 0.6528	0.5279	0.2057	0.1174 to 0.9600

## Data Availability

Data supporting the reported results may be provided upon reasonable request.
